# A Novel Nomogram Model Based on Cone-Beam CT Radiomics Analysis Technology for Predicting Radiation Pneumonitis in Esophageal Cancer Patients Undergoing Radiotherapy

**DOI:** 10.3389/fonc.2020.596013

**Published:** 2020-12-17

**Authors:** Feng Du, Ning Tang, Yuzhong Cui, Wei Wang, Yingjie Zhang, Zhenxiang Li, Jianbin Li

**Affiliations:** ^1^ Department of Radiation Oncology, School of Clinical Medicine, Cheeloo College of Medicine, Shandong University, Jinan, China; ^2^ Department of Radiation Oncology, Zibo Municipal Hospital, Zibo, China; ^3^ Department of Radiation Oncology, Shandong Cancer Hospital and Institute, Shandong First Medical University and Shandong Academy of Medical Sciences, Jinan, China

**Keywords:** esophageal cancer, cone beam computed tomography, radiation pneumonitis, prediction model, radiomics

## Abstract

**Purpose:**

We quantitatively analyzed the characteristics of cone-beam computed tomography (CBCT) radiomics in different periods during radiotherapy (RT) and then built a novel nomogram model integrating clinical features and dosimetric parameters for predicting radiation pneumonitis (RP) in patients with esophageal squamous cell carcinoma (ESCC).

**Methods:**

At our institute, a retrospective study was conducted on 96 ESCC patients for whom we had complete clinical feature and dosimetric parameter data. CBCT images of each patient in three different periods of RT were obtained, the images were segmented using both lungs as the region of interest (ROI), and 851 image features were extracted. The least absolute shrinkage selection operator (LASSO) was applied to identify candidate radiomics features, and logistic regression analyses were applied to construct the rad-score. The optimal period for the rad-score, clinical features, and dosimetric parameters were selected to construct the nomogram model and then the receiver operating characteristic (ROC) curve was used to evaluate the prediction capacity of the model. Calibration curves and decision curves were used to demonstrate the discriminatory and clinical benefit ratios, respectively.

**Results:**

The relative volume of total lung treated with ≥5 Gy (V5), mean lung dose (MLD), and tumor stage were independent predictors of RP and were finally incorporated into the nomogram. When the three time periods were modeled, the first period was better than the others. In the primary cohort, the area under the ROC curve (AUC) was 0.700 (95% confidence interval (CI) 0.568–0.832), and in the independent validation cohort, the AUC was 0.765 (95% CI 0.588–0.941). In the nomogram model that integrates clinical features and dosimetric parameters, the AUC in the primary cohort was 0.836 (95% CI 0.700–0.918), and the AUC in the validation cohort was 0.905 (95% CI 0.799–1.000). The nomogram model exhibits excellent performance. Calibration curves indicate a favorable consistency between the nomogram prediction and the actual outcomes. The decision curve exhibits satisfactory clinical utility.

**Conclusion:**

The radiomics model based on early lung CBCT is a potentially valuable tool for predicting RP. V5, MLD, and tumor stage have certain predictive effects for RP. The developed nomogram model has a better prediction ability than any of the other predictors and can be used as a quantitative model to predict RP.

## Introduction

Among malignant tumors, the incidence rate of esophageal cancer (EC) is the seventh highest, and the mortality rate is sixth worldwide (1). Radiotherapy (RT) is still one of the main treatments for locally advanced EC (2, 3). However, radiation pneumonitis (RP) is one of the major toxicities of thoracic radiation therapy. If RP occurs, it seriously affects the patient’s quality of life and survival prognosis (4). Therefore, it is imperative for EC patients undergoing RT to identify this toxicity at the earliest possible time. More importantly, the accurate prediction of RP is essential to facilitate individualized radiation dosing that leads to maximized therapeutic gain. At present, the risk assessment of RP is mainly predicted by using lung dosimetric parameters (5, 6), such as the relative volume of total lung irradiated above a specified threshold dose (V_X_) or mean lung dose (MLD): Although several metrics have appeared promising, the results vary across institutions, so these metrics do not seem to be perfect at predicting RP (7, 8). In addition to dosimetric parameters, some clinical features (tumor stage, smoking history, preexisting lung diseases, concurrent chemotherapy, and radiation dose) are also considered to be related to RP occurrence. However, the consensus on the comparative importance of these related predictors remains unavailable at present. Consequently, in order to individually and precisely discern RP, an accurate predictive model incorporating multiple types of factors with superior clinical utility is urgently needed.

Computed tomography (CT) images play an essential role in the diagnosis and treatment of RP. As early as the end of the 20th century, RP could be identified by CT. However, RP cannot be predicted by superficial CT manifestations. Therefore, the focus of later research is on the accurate prediction of RP (9). In recent years, with the rapid development of radiomics analysis technology, increasing attention has been paid to the research of RT effect and side effect predictions based on radiomics features (10–13). Among them, one study found that there is a dose-dependent relationship between the changes in some radiomics features and RP ≥2 grade determined by extracting local lung CT images after RT (12). Another study successfully established a differential model of high- and low-risk RP by analyzing the region of interest (ROI) of the whole lung tissue before RT (13). In short, radiomics features can capture the capability of lung texture features, which help describe the potential RP risk (14, 15).

At present, cone-beam computed tomography (CBCT) has become a routine online method of image-guided radiotherapy (IGRT) for EC. If we can perform quantitative analysis on CBCT radiomics features in a certain period of RT and then combine these radiomics features with clinical features and dosimetric parameters to predict RP in EC, it will help guide clinical treatment strategies in a timely manner.

Therefore, the initial aim of this study was to investigate whether the early changes in CBCT radiomics features could be used as potential markers for predicting RP. In the present study, a comprehensive nomogram, which is a conveniently applicable predictive model integrating CBCT radiomics features, clinical features, and dosimetric parameters, was built for the individualized risk assessment and precise prediction of RP.

## Materials and Methods

### Patients

The entire cohort of this retrospective study was obtained from the records of our institutional picture archiving and communication system (PACS) from January 2017 to June 2019, which was used to identify esophageal squamous cell carcinoma (ESCC) patients receiving RT treatment. The inclusion criteria were as follows: (1) Karnofsky performance score (KPS) ≥70, (2) no previous history of thoracic RT, (3) intensity-modulated radiotherapy (IMRT) and received ≥50 Gy RT, and (4) CBCT scan performed at least once a week during RT with the scanning range of the CBCT imaging including at least two thirds of the lungs. The exclusion criteria were as follows: (1) low image quality, (2) general pulmonary infection unrelated to RT, and (3) treatment break of more than 7 days during RT. A total of 96 consecutive patients with thoracic middle segment ESCC were identified and divided into two cohorts at a 7:3 proportion using computer-generated random numbers. Sixty-seven patients were allocated to the primary cohort, and 29 patients were allocated to the verification cohort. Our institutional research ethics board approved this retrospective study (SDTHEC201703014). It waived the need to obtain informed consent from the patients due to the retrospective nature of the investigation (retrospective single-institution cohort study).

### Clinical Data and RT Parameters

The clinical data were all acquired from the institute’s medical records. Specifically, clinicopathological parameters included age, sex, KPS, smoking status, diabetes history, chronic obstructive lung disease (COPD), pathological diagnosis, tumor location, TNM stage, radiation dose, and concurrent chemoradiotherapy lack thereof. In addition, the lung dosimetric parameters involved in this study included V5–V40 (relative volume of total lung treated with ≥5–40 Gy) and MLD. In short, the parameters mentioned above were used to establish a comprehensive nomogram after univariate analysis or least absolute shrinkage selection operator (LASSO) feature selection.

The Eclipse Treatment Planning System (Varian Medical Systems, Palo Alto, CA, Version 13.5.35) was adopted for RT planning design. IMRT adopts a fixed-field, static intensity modulation technique, and 5–7 fields of coplanar irradiation are uniformly divided according to the specific situation in each case. The required target parameters are then set, and the dose distribution is obtained by inverse calculation of the treatment planning system. The dose distribution is then graded (stratified), and each field is decomposed into a series of subfields. IMRT does not include sIMRT or volumetric intensity-modulated arc therapy (VMAT). The target area includes tumor volume (GTV), including CT imaging of visible esophageal tumors and positive lymph nodes. The clinical target volume (CTV) refers to the upper and lower expansion of the esophageal tumor by 3 cm and 6 mm around the tumor and related lymphatic drainage area. The planned target volume (PTV) is formed by CTV extending 8 mm outward. IMRT was administered by a Varian Linac Accelerator with a 6-MVX ray and 95% PTV, and radiation doses of 50–66 Gy (median dose of 60 Gy) and 1.8–2.0 Gy/fraction 5 times/week were prescribed.

Normal tissue constraints were prioritized in the following order for treatment planning purposes: maximum spinal cord dose of 45 Gy, relative volume of total lung treated with ≥5 Gy (V5) ≤60%, relative volume of total lung treated with ≥20 Gy (V20) ≤28%, MLD ≤20 Gy, relative volume of the heart treated with ≥30 Gy (V30) ≤40%, and relative volume of the heart treated with ≥40 Gy (V40) ≤30%.

### Follow-up and Evaluation of RP

Follow-up items included chest CT, physical examination, and clinical symptoms. Patients were evaluated weekly during RT, followed up at 1 month after completion of the initial treatment, and then followed up every 2–3 months until at least 6 months after the end of RT. The grading of RP was confirmed by two senior oncologists and one radiologist. The National Cancer Institute Common Terminology Criteria for Adverse Events 4.03 (CTCAE 4.03) was used to evaluate the degree of RP. In the present study, grade ≥2 was used as the cutoff for diagnosing RP.

### CBCT Scanning Method and Image Acquisition

Using the on-board imager (OBI) system mounted on the Varian Trilogy medical linear accelerator, the hardware portion included a diagnostic (kV) level X-ray source (KVS) and an amorphous silicon flat-panel kV detector (KVD). The CBCT image was obtained by rotating the frame at an angle. This is a slow CBCT acquisition setting. The acquisition time is 67 s, and the patient keeps breathing evenly during this process. Standard body scan conditions were voltage (125 kVp), current (80 mA), exposure time (13 ms), exposure (680 mAs), rotation angle (178°–182°), pixel matrix size (384×384), field of view (FOV, 45×18 cm), slice thickness (2.5 mm), and fan-beam type (half-fan). Among fan-beam types, the half-fan mode was used for the image acquisition of lung tissue structures larger than 24 cm. In this study, lung CBCT image acquisition was carried out in three different periods, and then the images were imported into 3D Slicer (version 4.10.2; http://www.slicer.org) in a DICOM format to extract and analyze the radiomics features. It should be noted that these three different periods were artificially divided according to the experimental design and corresponded to the early stages: the third, fourth, and fifth weeks of RT (PTV prescription dose range of EC: 18–22 Gy, 27–32 Gy, and 36–44 Gy).

### Image Segmentation and Feature Extraction

Images from both lungs were segmented by a semiautomatic segmentation method (16, 17) based on a threshold-based algorithm. The specific steps are as follows: First, the background was removed to obtain the internal region of the chest. Second, the appropriate threshold was found to segment the lung and the tissues outside the lung contour to the greatest extent. Finally, the manual segmentation method (18) was used to erase the extra parts outside the large trachea and lung parenchyma to obtain both lungs as the ROI. Image segmentation was performed by an experienced radiologist and then verified by a senior radiologist. All features were extracted by using the radiomics plug-in in 3D Slicer. A total of 851 radiomics features were extracted, including 13 morphological features, 18 histogram features, 74 original texture features, and 746 high-order features (wavelet transform features).

### Radiomics Feature Selection and Radiomics Signature Construction

First, the extracted radiomics features were preprocessed. Based on the Spearman rank correlation test, the features with correlations greater than 0.9 and multicollinearity were deleted, and independent features were preliminarily screened. Meanwhile, based on the Mann–Whitney *U* test, the characteristics with significant differences between the RP (≥2 grade) and non-RP (<2 grade) groups were screened out. Finally, the LASSO method (19) was used to select the final features, and the RP prediction model of rad-score was constructed based on logistic regression analysis. The LASSO method minimizes the sum of squared residuals by using the case in which the sum of the absolute values of the coefficients is less than the tuning parameter (λ). To prevent overfitting of the model, (20–22) during model building, features are selected by constantly adjusting λ. With the increasing penalty, more regression coefficients are reduced to zero, (23, 24) and then the remaining nonzero coefficient is selected. The nonzero coefficient of the selected features is the rad-score. Each patient’s rad-score is calculated as a linear combination of selected features that have their own coefficient weighting.

In this study, 50 iterations of 10-fold nested cross-validation were utilized, similarly to Xu et al. (25). Random sampling was conducted in an attempt to balance the class distributions within the cross-validation partitions. The cross-validation loop provides a profile of model performance. It serves to estimate how well the LASSO applied to a given set of candidate predictors may generalize to other data sets. Model performance was assessed by computing the area under the curve (AUC) for each constructed model on a test partition. The inner cross-validation loop was applied to determine the optimal value for λ such that the resulting model was guarded against overfitting. The value of λ for each cross-validation partition was selected by determining the value that produced the most regularized model such that the AUC was within one standard error of the maximum (26). The use of 50 resampled iterations with 10-fold nested cross-validation constructs 500 models used to generate a distribution of AUC values to estimate how well model construction with LASSO generalizes to other data sets.

### Construction and Validation of the Nomogram

First, the prediction efficiency of the three different periods was compared, and then the best period was selected. Second, 96 patients were divided into the RP (39 cases) and non-RP (57 cases) groups, and 16 clinical features and dosimetric parameters were collected. The best clinical features and dosimetric parameters were determined by LASSO feature selection. Finally, a comprehensive nomogram was established. The receiver operating characteristic (ROC) curve was used to evaluate the prediction capacity of the model. The calibration curve was used to determine whether the predicted and observed probabilities for RP were in concordance. The decision curve was performed to evaluate the clinical benefit ratio of the nomogram.

**Figure 1:**
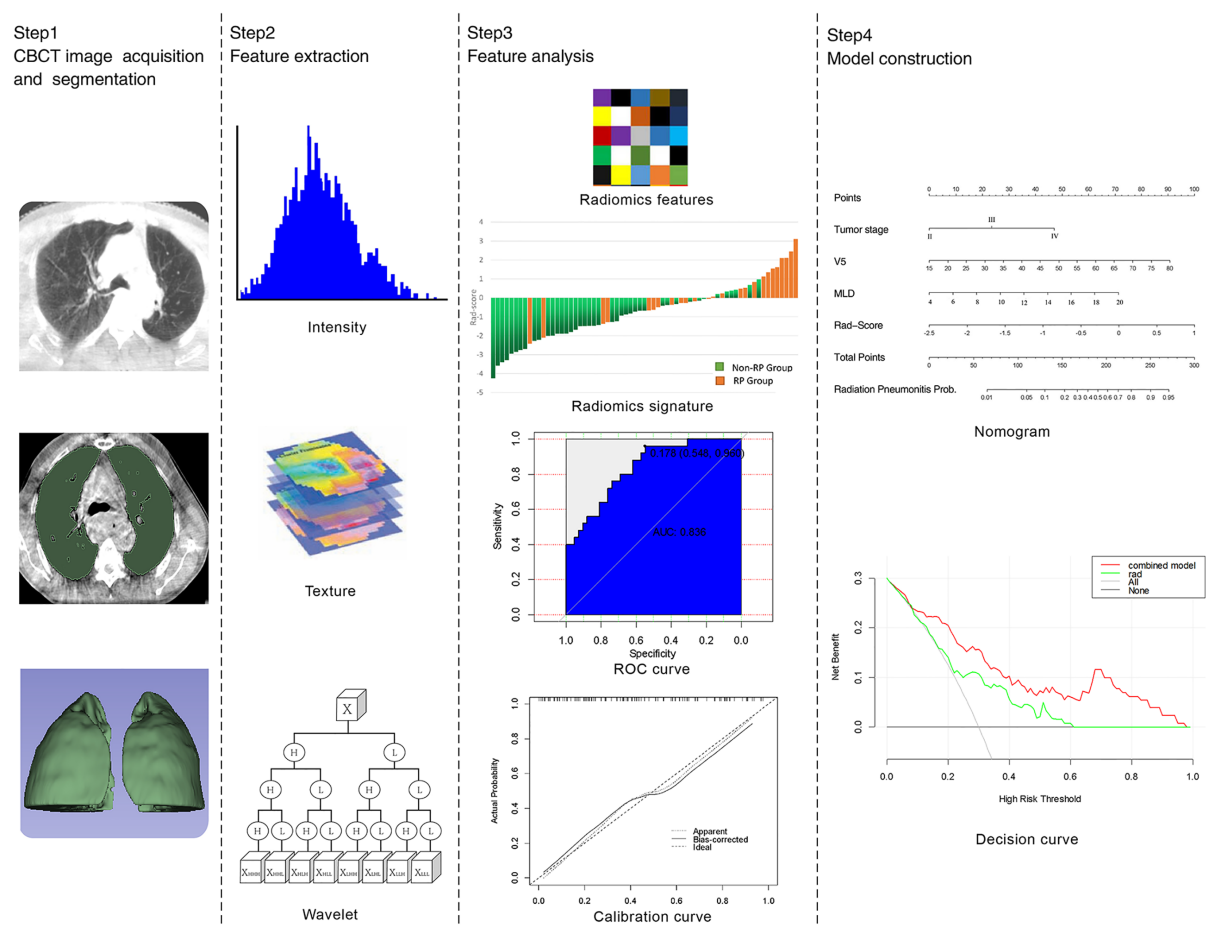
Flow chart of radiomics.

This research process can be divided into four parts: image acquisition, ROI segmentation, feature extraction, and radiomics model construction as shown in **Figure 1**.

### Statistical Analysis

All statistical analyses were based on SPSS 20.0 (IBM, Armonk, NY, USA) or R software (R Foundation for Statistical Computing, Vienna, Austria, https://www.R-project.org/). The χ^2^ test or Fisher exact probability test was used to classify data between the two groups. Two independent-sample *t* tests were used for counting data (continuous data). The Mann–Whitney *U* test was used to compare the differences in clinical features between the primary and validation cohorts. The model was evaluated with respect to sensitivity, specificity, ROC curve, and 95% confidence interval (CI). *P* values ≤ 0.05 were considered statistically significant.

## Results

### Analysis of Clinical Features and Dosimetric Parameters Associated With RP

The 96 patients were divided into RP (39 cases) and non-RP (57 cases) groups, and 9 clinical features and 7 dosimetric parameters that might be related to the occurrence of RP were included. Univariate analysis showed that tumor stage was correlated with ≥2 grade RP (χ^2^ = 2.650, *P* = 0.008), and other factors, including age, sex, concurrent chemoradiotherapy or lack thereof, COPD status, smoking status, and RT dose, showed no significant differences between the two groups (all *P*s > 0.05). V5, V10, V15, V20, V30, and MLD of both lungs were associated with the occurrence of grade ≥2 RP (all *P*s < 0.05). The characteristics of the enrolled population are listed in **Tables 1** and **2**.

**Table 1:** Univariate analysis of baseline clinical features of patients and RP.

Factor	*N*	RP		χ^2^ value	*P* value
		<2 grade	≥2 grade		
**Sex**	96	57	39	2.767	0.096
Male	81	51	30		
Female	15	6	9		
**Age (years)**	96			1.619	0.203
<60	21	15	6		
≥60	75	42	33		
**Stage**				2.650	0.008
II	19	15	4		
III	48	30	18		
IV	29	12	17		
**Smoking history**	96			0.198	0.656
No	54	31	23		
Yes	42	26	16		
**COPD**	96			1.436	0.231
No	81	46	35		
Yes	15	11	4		
**Diabetes**	96			0.318	0.573
No	88	53	35		
Yes	8	4	4		
**Hypertension**	96			0.606	0.436
No	83	48	35		
Yes	13	9	4		
**Concurrent** **Chemotherapy**	96				
No	71	41	30	0.300	0.584
Yes	25	16	9		
**Delivered** **Dose (Gy)**	96			1.867	0.172
<60	45	30	15		
≥60	51	27	24		

COPD, chronic obstructive lung disease.

**Table 2:** Single factor analysis of DVH and RP.

Lung DVH	RP		*P* value	χ^2^ value
	<2 grade	≥2 grade		
**V5**	48.95 ± 10.56	59.39 ± 10.00	0.00	-4.91
**V10**	33.64 ± 7.70	40.92 ± 7.95	0.00	-4.46
**V15**	25.34 ± 6.52	30.77 ± 6.96	0.00	-3.85
**V20**	18.81 ± 5.47	22.47 ± 4.82	0.00	-3.47
**V30**	9.61 ± 4.40	12.16 ± 5.00	0.01	-2.58
**V40**	3.80 ± 2.49	4.58 ± 3.24	0.21	-1.25
**MLD (cGy)**	1016.47 ± 218.82	1260.87 ± 267.38	0.00	-4.72

MLD, mean lung dose; V5, V10, V15, V20, V30, V40 = relative volume of total lung treated with ≥5, 10, 15, 20, 30, and 40 Gy.

**Figure 2:**
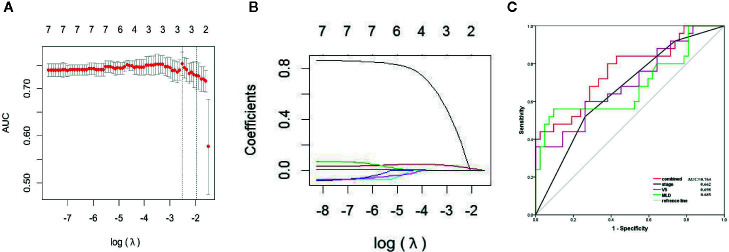
LASSO characteristic selection of clinical features and dosimetric parameters **(A, B)**. ROC curve of V5, MLD, tumor stage, and combined model **(C)**.

There were no significant differences in age, sex, tumor stage, V5, and MLD between the primary group and the validation group, which indicates that the groupings were reasonable (all *P*s > 0.05) as shown in **Table 3**. Seven factors (tumor stage, V5, V10, V15, V20, V30, and MLD) remained after univariate analysis. The LASSO feature selection method was used to screen these seven factors, and three potential factors (V5, MLD, and tumor stage) remained as shown in **Figures 2A, B**. The AUC values of prediction efficiency for V5, MLD, and tumor stage were 0.698, 0.685, and 0.662, respectively. To observe the overall predictive performance of V5, MLD, and tumor stage, we established a full clinical–dosimetric feature combined model. The AUC value of the combined model was 0.764 as shown in **Figure 2C**.

**Table 3:** Comparison of sex, age, tumor stage, V5, and MLD between the primary and the verification cohort.

Factor	Primary cohort	Verification cohort	χ^2^ value	*P* value
**Age (years)**	65.33 ± 9.37	68.62 ± 8.89	-1.64	0.11
**Sex (*N*)**	67	29	0.11	0.75
Male	56	25		
Female	11	4		
**Stage**			3.54	0.17
II	13	6		
III	30	18		
IV	24	5		
**V5**	52.35 ± 11.27	55.14 ± 12.01	-1.07	0.29
**MLD (Gy)**	11.06 ± 2.61	11.38 ± 2.85	-0.52	0.61

### Radiomics Feature Extraction/Selection at Different Periods and Radiomics Signature Building

**Figure 3:**
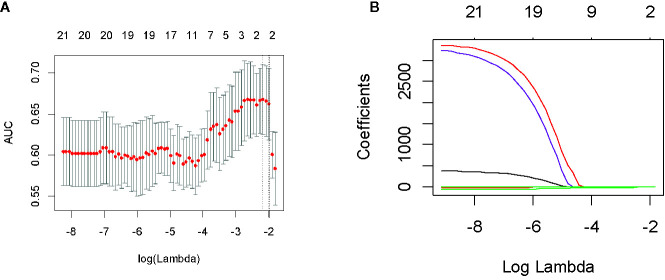
Feature screening of radiomics in the first period. By adjusting the different penalty parameter (λ) to obtain a high-performance model, the radiomics characteristics with the highest predictive performance were obtained. Radiomics feature convergence diagram **(A)**. Each curve represents the trajectory of the coefficient of each independent variable **(B)**.

**Figure 4:**
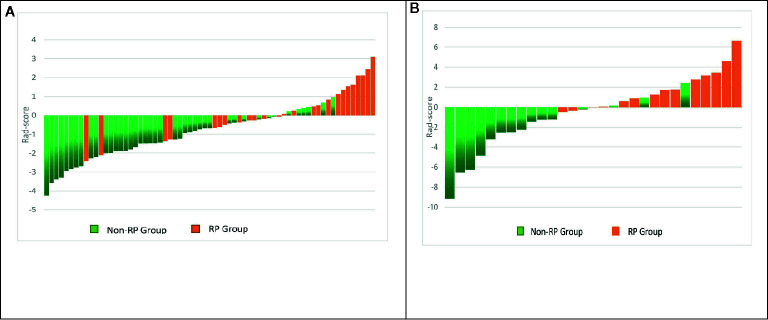
Rad-score for each patient in the primary and validation cohorts. Green bars show scores for patients without RP, and orange bars show scores for those with RP **(A, B)**.

In the first period (PTV dose: 18–22 Gy), a total of 851 radiomics features were extracted from the patients. First, correlations greater than 0.9 features were deleted, resulting in a total of 220 features remaining. Second, linear features were removed, and 96 features remained. Then, 21 features remained after using the rank-sum test. Finally, the remaining two features after LASSO selection were used to build the radiomics model as shown in **Figures 3A, B**. The two features are original first-order skewness and original GLSZM-small area emphasis. The model was built as follows: Rad-score = -0.924 e+00×Skewness - 7.047 e+00×Small Area Emphasis + 4.5329. Rad-scores for each patient in the primary cohort and validation cohort are shown in **Figures 4A, B**.

In the second period (PTV dose: 27–32 Gy), a total of 851 radiomics features were extracted from the patients. First, correlations greater than 0.9 features were deleted, resulting in a total of 222 features remaining. Second, linear features were removed, and 96 features remained. Then, 10 features remained after using the rank-sum test. Finally, the remaining five features (voxel volume, smallest axis length, small dependence low gray-level emphasis, large area low gray-level emphasis, and busyness) after LASSO selection were used to build the radiomics model. The model was built as follows: Rad-score = -1.996 e-07×voxel volume - 4.036 e-03×smallest axis length + 5.376 e+01×small dependence low gray-level emphasis + 1.718 e-07×large area low gray-level emphasis - 2.473 e-04×busyness + 1.041 e+00.

In the third period (PTV dose: 36–44 Gy), a total of 851 radiomics features were extracted from the patients. First, correlations greater than 0.9 features were deleted, resulting in a total of 220 features remaining. Second, linear features were removed, and 96 features remained. Then, 43 features remained after using the rank-sum test. Finally, the remaining six features (gray-level nonuniformity, small dependence low gray-level emphasis, cluster shape, uniformity, entropy, and size zone nonuniformity) after LASSO selection were used to build the radiomics model. The model was built as follows: Rad-score = +4.680 e-07×gray-level nonuniformity + 1.087 e+01×small dependence low gray-level emphasis - 7.913 e-04×cluster shape + 1.401 e+00×uniformity + 1.406 e+00×entropy - 2.207 e-05×size zone nonuniformity - 4.776 e+00.

### Validation of Radiomics Signature at Different Periods

**Figure 5:**
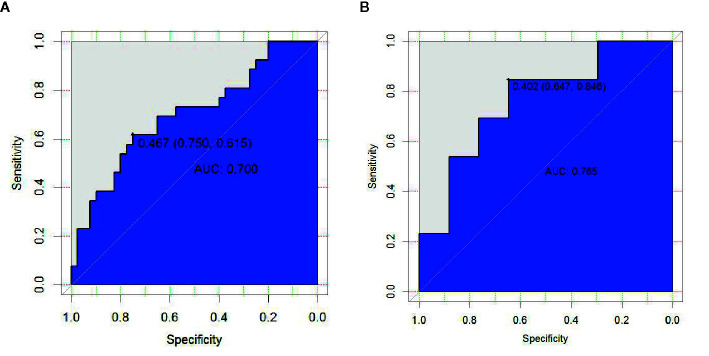
ROC curve of radiomics features in the first period of RT **(A, B)**.

In the first period, the predictive efficacy of the model was as follows: In the primary cohort, the AUC was 0.700 (95% CI 0.568–0.832), the sensitivity was 61.5%, and the specificity was 75.0%. In the validation cohort, the AUC was 0.765 (95% CI 0.588–0.941), the sensitivity was 84.6%, and the specificity was 64.7% as shown in **Table 4** and **Figures 5A, B**.

**Table 4:** ROC curve parameters of the radiomics model and nomogram.

Classification	Primary cohort				Validation cohort			
	AUC	95% CI	Sensitivity	Specificity	AUC	95% CI	Sensitivity	Specificity
First period	0.700	0.568-0.832	61.5%	75.0%	0.765	0.588-0.941	84.6%	64.7%
Second period	0.663	0.530-0.797	90.6%	42.9%	0.604	0.356-0.851	85.7%	50.0%
Third period	0.699	0.573-0.826	66.7%	70.3%	0.756	0.561-0.950	66.7%	80.0%
Nomogram	0.836	0.700-0.918	96.0%	54.8%	0.905	0.799-1.000	92.9%	73.3%

In the second period, the predictive efficacy of the model was as follows: In the primary cohort, the AUC was 0.663 (95% CI 0.530–0.797), the sensitivity was 90.6%, and the specificity was 42.9%. In the validation cohort, the AUC was 0.604 (95% CI 0.356–0.851), the sensitivity was 85.7%, and the specificity 50.0%.

In the third period, the predictive efficacy of the model was as follows: In the primary cohort, the AUC was 0.699 (95% CI 0.573–0.826), the sensitivity was 66.7%, and the specificity was 70.3%. In the validation cohort, the AUC was 0.756 (95% CI 0.561–0.950), the sensitivity was 66.7%, and the specificity was 80.0% as shown in **Table 4**.

By comparing the prediction efficiency of the AUC in three periods, it is obvious that the prediction efficiency in the first period is better than those in the second and third periods in both the primary and validation cohorts. To reflect the importance of the early prediction of RP in clinical practice, the first-period rad-score and three essential features (V5, MLD, and tumor stage) were used to establish a comprehensive nomogram model.

### The Incremental Value of the Radiomics Signature When Added to the Comprehensive Nomogram

**Figure 6:**
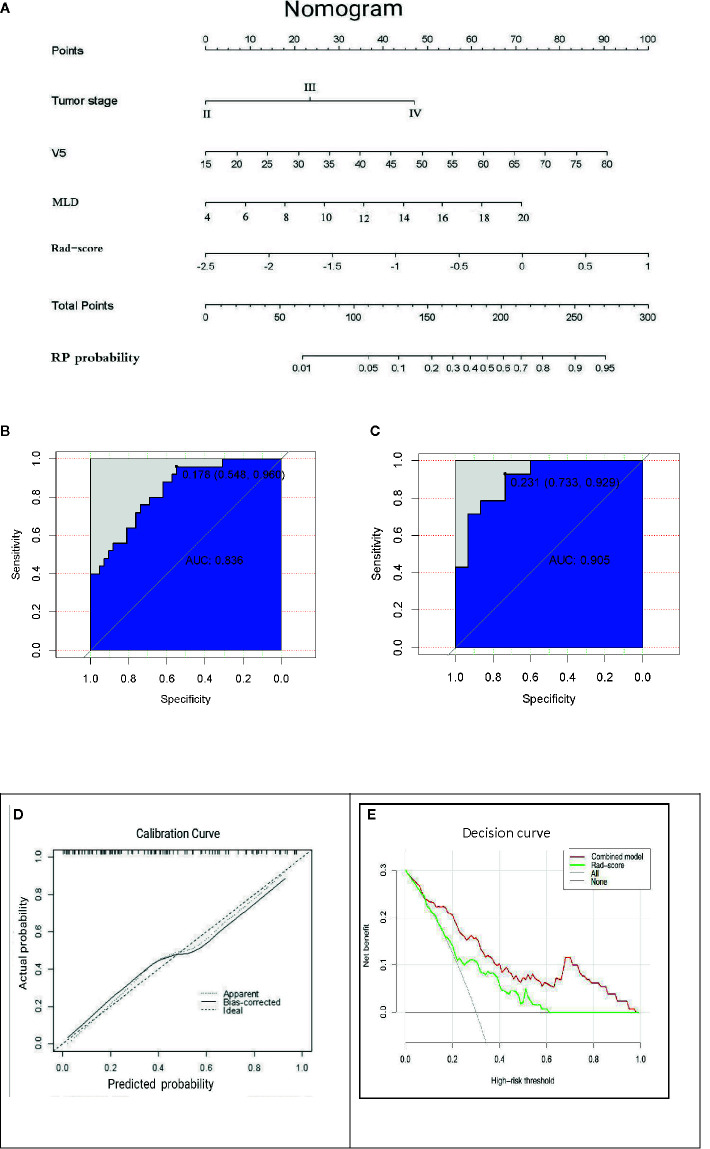
**(A)** The comprehensive nomogram incorporates V5, MLD, tumor stage, and rad-score (the first period) to predict the risk of RP in EC patients. V5: relative volume of total lung treated with ≥5 Gy; MLD: mean lung dose. **(B, C)** ROC curves of the comprehensive nomogram in the primary and validation cohorts. **(D)** Calibration curves of the comprehensive nomogram with the addition of V5, MLD, tumor stage, and radiomics features. The diagonal dotted line represents an ideal evaluation, and the other two lines next to it represent the performance of the nomogram. A closer fit to the diagonal dotted line indicates a better evaluation. **(E)** Decision curves of the radiomics features model and the combination model (comprehensive nomogram) predicting RP. The *y*-axis represents the net benefit. The red curve represents the comprehensive nomogram, and the green line represents the radiomics features model. The horizontal black line indicates that the assumption is valid. The oblique gray line indicates that the assumption is invalid.

The AUC values of dosimetric parameters (V5, MLD) and clinical features (tumor stage) were 0.698, 0.685, and 0.662, respectively. The AUC values of the full clinical–dosimetric feature combined model was 0.764. In addition, the AUC values of the radiomics signature at three different periods were 0.700, 0.663, and 0.699, respectively (primary cohort). It can be seen that the single clinical features, dosimetric parameters, or full clinical–dosimetric combined model are not ideal in predicting the risk of RP. To this end, we created a comprehensive nomogram that integrates dosimetric parameters and clinical features with the radiomics signature from the first period. The results show that, in the primary cohort, the AUC of our nomogram was 0.836 (95% CI: 0.700–0.918), and in the validation cohort, the AUC was 0.905 (95% CI: 0.799–1.000) as shown in **Table 4** and **Figures 6B, C**. There is no doubt that the comprehensive nomogram, incorporating radiomics features, significantly improves the ability of conventional dosimetric parameters and clinical features to predict the risk of RP. The graphical form of the nomogram is shown in **Figure 6A**. More importantly, the calibration curve is produced as shown in **Figure 6D**. The diagonal dotted line represents an ideal evaluation, and the other two lines next to it represent the performance of the nomogram. A closer fit to the diagonal dotted line indicates a better evaluation. In summary, this calibration curve shows favorable consistency between the predicted RP and the actual observation.

### How to Make Clinical Decisions

The clinical decision curve analysis of the nomogram is shown in **Figure 6E**, which shows the patient’s benefits when the physician makes the judgment. It shows that, if the probability of the domain value is 10%, the benefit of using the nomogram to predict the efficacy of RP is higher than that of radiomics features or other parameters alone. In short, this decision curve exhibits satisfactory positive net benefits of the nomogram at the threshold probabilities.

## Discussion

A single index based on lung dosimetric parameters is not the “gold standard” to judge the occurrence of PR risk; however, radiomics can extract image data to characterize the standard tissue structure, including typical lung structures. They may produce clinically relevant improvements in predicting treatment-related toxicities (13). This makes up for the deficiency of dose-volume parameter prediction to a great extent. Some previous studies, (12, 13) respectively, report the relationship between the changes in some second- or higher-order eigenvalues of lung cancer after and before RT and the occurrence of RP. Unfortunately, due to the limitations of detection techniques or other factors, it is not possible to establish predictive models for clinical practice. In this study, we used an automated computer extraction algorithm and digital quantitative analysis technology to obtain high-quality information to comprehensively evaluate various characteristics of tumor and normal tissue responses (14, 27). More importantly, we constructed a comprehensive nomogram model based on CBCT radiomics features in combination with clinical features and dosimetric parameters to accurately predict RP in EC patients treated with RT. To the best of our knowledge, this is the first study of the early prediction of RP by using IGRT to obtain CBCT imaging information in different periods of RT. Importantly, this comprehensive nomogram model is superior to single clinical features and lung dosimetric parameters in RP prediction.

We selected CBCT images from three different periods and extracted the radiomics features. The primary purpose was to find the first radiomics features that can independently predict RP; however, after selecting the radiomics features in different periods, it is found that each period has its own independent set of feature parameters related to RP. We believe that, in addition to the influence of radiation dose factors, whether these characteristics vary with changes in the RT process is still uncertain. It is gratifying that we found the best prediction of RP to be in the first period of radiomics characteristics. Two important features can be found in the early stage of low-dose RT of lung tissue: Although this may differ from our initial expectation of the experimental results, the results are fascinating. This result is similar to the findings of Cunliffe et al. (12) and Jenkins et al (28). They found that AUC values in low- and medium-dose areas of the lung were different between RP and non-RP patients even though these AUC values appeared in areas with lower visible changes. These first radiomics features may be able to be used to explain or screen out those susceptible to RP due to intrinsic genetic mutations.

In regard to the susceptible population of RP, we must devote attention to the sensitivity of lung tissue to RT. At present, the radiosensitivity of lung tissue has been reported (29, 30), and it is considered to be a potential influencing factor for RP occurrence. This difference in the sensitivity of lung tissue to radiation constitutes our different understanding of the probability of RP. In two groups of patients with different radiosensitivity of lung tissue, we cannot judge the probability of RP by standard clinical features and lung dosimetric parameters. However, radiomics can analyze the data by extracting features from CT images of the lung, thus providing a powerful method for the noninvasive description of lung tissue radiosensitivity. This may be why the radiomics features are superior to the clinical features and dosimetric parameters in current studies. In this study, this advantage in AUC value, sensitivity, and specificity performance is not particularly good, but through our research analysis, radiomics features of RP risk prediction cannot be ignored.

The successful establishment of the prediction model is based on the standardization of data collection and the rationalization of data processing. First, we should consider that the feature extraction data are affected by CT parameters (31) because the CT features may be different under different image-acquisition conditions. In this study, based on the CBCT of the Varian accelerators in our center, these devices have the same tube voltage, tube current, exposure time, exposure amount, and pixel matrix size, which can help control for the differences between the scanners and acquisition parameters. Second, to develop the radiomics signature, a total of 851 candidate features were reduced to a set of only a few potential descriptors by using the LASSO logistic regression model to realize feature selection by constantly adjusting the regularization parameter λ to make the weight coefficient of the feature approach zero. The LASSO (20) logistic regression model is suitable for analyzing large sets of radiomics features with a relatively small sample size, and it is designed to avoid overfitting high-dimensional data (21, 32). At the same time, the LASSO logistic regression model allows the radiomics signature to be constructed by combining the selected features, so it allows the model to more easily identify the most closely related features in patients with RP. Finally, the nested cross-validation method (25) was used for internal validation to improve the accuracy of the model.

It should be noted that the difference in the irradiation mode (3-D conformal radiation therapy and IMRT) affects the potential dose distribution of the lung, which may affect the selection of clinical features and dosimetric parameters as risk characteristics of RP. This can be quickly confirmed by comparing Tucker et al. (33) and Shane et al. (13) where, in the former, 75% received 3-D conformal radiation therapy, and the latter 83% received IMRT. Therefore, it seems complicated to establish a general model with good discriminant performance under different technical conditions.

The clinical factors (age, tumor stage, KPS score, chronic lung disease, diabetes, chemotherapy lack thereof) and lung dosimetric parameters (V5, V10, V20, MLD) related to RP are reported in previous studies. To provide better help for the oncologist, we designed a clinical nomogram to combine the above available RP risk factors with radiomics features. Therefore, we aim to establish a combined model, maximizing clinical utility and accuracy of prediction ability, and so the initial experimental design was not expected to rely solely on the radiomics model as the final prediction model. Of course, judging from the AUC value, sensitivity, and specificity of the radiomics model in each period of RT, these characteristics alone are not perfect in predicting RP. Dose-volume histogram (DVH) metrics have been extensively observed and reported to be correlated with RP despite the current data and research reports not being sufficient to provide specific and safe standard doses (34). Chargari et al. (35) find that V5 is a risk factor for acute or chronic lung toxicity. Cho et al. (6) find that MLD is the most related factor that predicts RP rather than V5, V10, or V20. Some clinical features have emerged as important risk factors contributing to RP progression. Some studies show that smoking is related to the severity of RP (36, 37). Takeda et al. (38) and Kimura et al. (39) report that COPD is a significant risk factor for RP in patients with EC after RT. In this study, we find that smoking status, COPD, and concurrent chemoradiotherapy are not correlated with the incidence of RP, and so these factors are not included in our combined model, but this does not mean that they are not important. After LASSO logistic regression analysis, several significant variables, including V5, MLD, and tumor stage, were integrated into the nomogram to predict PR. The results were as follows: clinical-dose characteristic model (AUC values: V5 = 0.698, MLD = 0.685, tumor stage = 0.662), radiomics model (primary cohort AUC 0.700, validation cohort AUC 0.765), and nomogram (primary cohort AUC 0.836, validation cohort AUC 0.905). The nomogram demonstrates a better ability to predict RP than the other models.

How to use this information in the treatment plan or alternative program to help clinicians is our greatest concern. Fortunately, the goal of radiomics is to develop a decision-making tool that meets the needs of clinicians. This is because such a tool could combine radiomics features with other patient characteristics to improve the capability of the decision support model (15, 40). We show that radiomics features complement clinical features and lung dosimetric parameters, helping to provide better predictive ability for RP. The clinical decision curve of this nomogram shows that the effectiveness of the nomogram in predicting RP is higher than that of using radiomic characteristics or other parameters alone. In short, under the threshold probability, the decision curve exhibits a satisfactory positive net benefit of the nomogram.

Our results demonstrate the potential value of radiomics techniques in the risk prediction of RP patients. If more clinical variables are included in the nomogram, there will be more room for future development of this model, and the resulting prediction effect will be better. A recent study (41) by another of our teams found that subjective global assessment score (SGA), pulmonary fibrosis score (PFS), planning target volume/total lung volume (PTV/LV), MLD, and ratio of change regarding systemic immune inflammation index at 4 weeks (4w SII) were potential valuable markers in predicting severe acute radiation pneumonitis (SARP). Subsequently, the team developed a nomogram and corresponding risk classification system with superior prediction ability for SARP. In the next step, we will consider combining the research results of this team with radiomics to establish a new RP prediction model for better clinical application.

Although our study has many strengths, several limitations should be addressed here. First, the sample size is small, which can lead to the inability to apply nonlinear classifiers, such as neural networks (42, 43). Because a nonlinear classifier uses a more extensive data set, it is beneficial to improve the accuracy of the RP model. Second, our analysis does not account for two-way or higher-order interactions between features. If interactions between features had been identified, the interaction terms that were most strongly associated with the outcome interactions would have been selected when we constructed the radiomics signature, and this could have improved performance. However, uncovering the interactions of multiple attributes is a challenging problem. Third, we used a validation cohort that was drawn from the same institution as the primary cohort, which prevented us from investigating the generalizability of the results to other institutions and settings. In addition, there is a lack of sufficient external data validation. Fourth, selection bias occurred when strict criteria were used, and this may affect the model training. For instance, all patients are middle thoracic EC patients, which limits the application of this method to patients with cervical, upper, and lower thoracic segment EC radiotherapy. Also, all patients experienced uniform CBCT imaging scanners and parameters, which does not guarantee the reproducibility and stability of radiomics features under other conditions. In the future, we should conduct a prospective, multicenter, large-cohort study to further develop and validate nomograms in terms of predicting RP.

As a future study, we will add different types of patients, including those with different EC locations (cervical, upper thoracic, lower thoracic segments) and different RT techniques (3DCRT, TOMO, VMAT). We will also include more laboratory indicators that may reflect RP, such as inflammatory indexes and immune inflammatory indexes. In terms of basic research, we should also improve the model of radiomics, especially the combination of radiomics and genomics. The former focuses on medical imaging of the normal tissues or tumors and performs diagnosis and prognosis based on quantitative imaging features, and the latter discovers and notes the gene sequences to study the function and structure of genomes of the diseases. Besides this, if we can combine available radiation metabolomics (44) with functional CT (45, 46) radiomics features, it may help us understand the differences in radiation sensitivity and tissue cell metabolism in order to establish a more robust prediction model. Therefore, it can be predicted that the combination of multiple omics will be the best plan for the future diagnosis and treatment of diseases and the prediction of complications.

## Conclusions

CT radiomics has powerful data-processing and analysis abilities. In this context, we explored a method to predict RP based on the lung CBCT radiomics features for EC patients. More importantly, we used this method to successfully build and validate a novel nomogram with good predictive value, which can help clinicians identify high-risk RP patients early and guide personalized treatment and clinical decisions.

## Data Availability Statement

The raw data supporting the conclusions of this article will be made available by the authors, without undue reservation.

## Author Contributions

FD and NT are responsible for analyzing data and writing papers. JL designed experiments to guide the writing and revision of papers. WW directed the writing and revision of papers. YC, YZ, and ZL were responsible for radiomics diagnosis and radiomics data processing of patients. All authors contributed to the article and approved the submitted version.

## Funding

Funding was obtained from the National Key Research Program of China (No. 2016YFC0904700), the National Natural Science Foundation of China (No. 81773287), and The Key Research Development Program of Shandong Province (No. 2016GSF201093).

## Conflict of Interest

The authors declare that the research was conducted in the absence of any commercial or financial relationships that could be construed as a potential conflict of interest.
